# Matrons and Sisters

**Published:** 1916-02-05

**Authors:** 


### THE DEPARTMENT OF NURSING.

### Matrons and Sisters.

The war has brought many changes and created a multitude of difficulties, with greatly increased work to all members of the staff in voluntary hospitals and Poor-Law infirmaries. The same may be said of the war-created hospitals at home, which include Poor-Law infirmaries and lunatic asylums converted into Military hospitals, the Territorial hospitals, and converted buildings and houses of various kinds now used for hospital purposes, which include hospital accommodation for officers of all ranks. In every British hospital in 1915 the work which has devolved upon the matrons and sisters has been great and varied. It has demanded the exhibition on their part of keen intelligence, untiring energy, and supreme devotion to duty. We Britons have a right to be proud of our women workers, and to appreciate at its true value all that they have done, and are doing, through the hospitals for the wounded sailors and soldiers.

During the last fortnight we have had the privilege to receive a great number of letters from matrons who are in responsible charge of the hospitals throughout the country. The time has arrived when a definite and sustained attempt should be made to encourage every one of them, by quickening the interests of the public throughout the country in their work, that the people of all classes may appreciate the difficulties overcome and the indebtedness of the nation to these able and devoted administrators. We shall endeavour to bring out from week to week as clearly as possible the more striking features of the work, and to publish interesting information of the life in our hospitals under the conditions which war has created. We are promised the co-operation of many of those whose daily duties are discharged in the very heart of our hospital system, with a view to make the department of nursing and the service it renders to the nation apprehended, and so place it in a position to receive the full recognition which it merits.

Again, this section of The Hospital cannot fail to prove helpful to matrons and sisters everywhere. In our present issue we give some important information not easily obtainable on the subject of "Textile Supplies and Hospital Maintenance in Wartime." In a letter on the latter subject an expert gives some very interesting information as to the past and present cost of textiles, including linen, and the reasons which have contributed to increase that cost seriously. These are practical matters of great interest and importance. We invite every matron and sister to send us an inquiry upon any point of difficulty or doubt in any department of her work which may arise in the course of it. This may enable us some day to render practical service by lessening or removing a difficulty or in solving some problem of the moment.

To make this new departure in journalism as effective as possible it is essential that the opening thus afforded shall be used to the full by matrons and sisters. It has been our invariable practice to pay for all contributions which we may publish—that is, not only articles, but items of news. The latter should occupy an important place in the scheme we have just referred to, especially when they deal with topical subjects or current events. The minimum payment, if published, is 5s. There will be no hard-and-fast rule as to space, but paragraphs of about twenty lines in length—that is to say, of 150 words—are preferred. In this section during the war we propose to give 10s. to the correspondent who may send us in any week the best incident connected with hospital life and work. In this way matrons and sisters will, we hope, receive practical aid and encouragement; while those who reciprocate will be able to render us service by making it possible successfully to continue and develop the scheme we have here outlined. Contributions should be addressed to The Editor, The Hospital, 28 & 29 Southampton Street, Strand, London, W.C., and, to be in time for the current week's issue, should reach him by the first post on Mondays.

